# Country Phthisis

**Published:** 1900-11-24

**Authors:** 


### COUNTRY PHTHISIS.

Dr. Clifford Beale, writing in *Tuberculosis*, draws attention to the frequency with which tuberculous affections are met with in the Welsh and Scottish mountain districts, amid external surroundings which would seem at first sight to conduce in every way to the maintenance of the most vigorous health. He attributes this apparent anomaly to the closeness and lack of ventilation within the cottages, thus strongly supporting the position we took up in commenting on a recent paper read before the Royal Medical and Chirurgical Society on the influence of exposure to winds upon the prevalence of phthisis.

From observations made during many years he says that in very many of the high-lying cottages where the weather, during the colder months, was generally stormy, it was the exception to find a window that was capable of being opened, and no attempt was made, even in the hottest weather, to introduce more air than could find its way into the house by way of the door. Except in the case of a death in the house, it often happens that the furniture of the combined living and sleeping room remains undisturbed for years. He says, "I have seen cases of tubercular disease of the lung shut up in the closest of rooms in mountain cottages, every means taken to exclude air, the patient wrapped in flannel garments of the heaviest kind, saturated with sweat and dirt, and breathing an atmosphere overladen with human, and sometimes animal, exhalations, thickened—and, it is to be hoped, partially sterilised—by heat or coal smoke on stormy days," and he goes on to point out how great must be the risk of infection to the female attendants in such a case, and to the young children, who must needs be within doors during the many wet days of the year. We all nowadays recognise that tuberculosis is infectious, taking the word infectious with certain limitation. But everything points to the probability that it is by a house infection or by a workshop infection rather than by anything scattered abroad in the open that the disease is spread.

